# The Microbial Challenge: Human-Microbe Interactions

**DOI:** 10.3201/eid0812.020624

**Published:** 2002-12

**Authors:** Joseph E. McDade

**Affiliations:** Rome, Georgia

During the past decade, a wide array of books have addressed the topic of new and emerging infectious diseases. In The Microbial Challenge: Human-Microbe Interactions, Robert I. Krasner attempts to put this topic into a format appropriate for classroom presentation. The book is sufficiently well done to stand alone in some situations, but supplemental material may be necessary in others.

The Microbial Challenge is relatively compact, compared with the usual microbiology textbook, with expected advantages and unavoidable shortcomings. In 400 pages and 16 chapters, the author captures basic principles of microbiology, immunology, epidemiology, and infectious diseases, including chapters on biological weapons, “current plagues,” and factors underlying emergence of infectious diseases. A chapter on the control of microbial diseases is not unexpected, but the subsequent chapter on partnerships in the control of infectious diseases is an unusual and welcome addition. Chapters are organized into three major parts: “The Challenge,” “Meeting the Challenge,” and “Current Challenges.” However, these designations reflect the author’s perspective more than specific content. Similar chapters in other texts might simply be categorized under the generic theme “microorganisms and humans.”

The topics and organization in the book comprise characteristics well suited for a textbook. Each topic is presented clearly and succinctly. The book has many wonderful photographs and illustrations that bring the text to life, including unique photographs from field situations (many from the author’s personal archives). These photographs demonstrate that microbiology and infectious diseases extend far beyond the hospital and laboratory. A series of self-evaluation questions follow each chapter. Students will find the glossary quite useful. The author also provides a brief list of websites that might be helpful to instructors.

Although this book provides an informative summary of principles that are quite useful for a survey course on microbiology and infectious diseases, the abbreviated treatment might prove unsatisfactory for some instructional situations. For example, do not expect to find the detailed descriptions of chemical principles, central metabolic pathways, DNA replication, transcriptional control of gene function, or protein and cell-wall synthesis found in standard (and much larger!) textbooks. Taxonomy and diagnostic microbiology are only briefly discussed. The focus on microorganisms and diseases at the expense of such details may be an asset or a liability, depending on your perspective.

The author anticipates that the book would be suitable for a course without a laboratory component but suggests that certain exercises be added from other sources, as needed. The decision not to include a laboratory component is a critical departure point for the book’s utility. Although the text would be quite suitable in some curricula for a survey course on microorganisms and infectious diseases, the failure to include principles and applications of molecular biology seems a serious omission, especially for students who go on to graduate school and pursue careers in laboratory-based research. Indeed, the only surprise I had in reviewing the book was the lack of discussion of molecular techniques because they are commonly used to identify and subtype pathogens during epidemiologic investigations. 

The Microbial Challenge could well serve audiences beyond the undergraduate level. The text would be an excellent choice for schools of public health, provided that students are given supplemental readings and detailed case studies for analysis. Considering the current interest in infectious diseases and bioterrorism, this book would be a useful resource for government staff at the national and local levels. 

